# Complexes of Silver(I) Ions and Silver Phosphate Nanoparticles with Hyaluronic Acid and/or Chitosan as Promising Antimicrobial Agents for Vascular Grafts

**DOI:** 10.3390/ijms140713592

**Published:** 2013-06-28

**Authors:** Dagmar Chudobova, Lukas Nejdl, Jaromir Gumulec, Olga Krystofova, Miguel Angel Merlos Rodrigo, Jindrich Kynicky, Branislav Ruttkay-Nedecky, Pavel Kopel, Petr Babula, Vojtech Adam, Rene Kizek

**Affiliations:** 1 Department of Chemistry and Biochemistry, Faculty of Agronomy, Mendel University in Brno, Zemedelska 1, CZ-613 00 Brno, Czech Republic; E-Mails: dagmar.chudobova@centrum.cz (D.C.); lukasnejdl@gmail.com (L.N.); merlos19792003@hotmail.com (M.A.M.R.); brano.ruttkay@seznam.cz (B.R.-N.); paulko@centrum.cz (P.K.); vojtech.adam@mendelu.cz (V.A.); 2 Central European Institute of Technology, Brno University of Technology, Technicka 3058/10, CZ-616 00 Brno, Czech Republic; E-Mails: j.gumulec@gmail.com (J.G.); petr_babula@email.cz (P.B.); 3 Karel Englis College, Sujanovo nam. 356/1, CZ-602 00, Brno, Czech Republic; E-Mails: olga.krystofova@seznam.cz (O.K.); jindrak@email.cz (J.K.)

**Keywords:** polymers, antimicrobial activity, silver ions, silver phosphate nanoparticles, hyaluronic acid, chitosan

## Abstract

Polymers are currently widely used to replace a variety of natural materials with respect to their favourable physical and chemical properties, and due to their economic advantage. One of the most important branches of application of polymers is the production of different products for medical use. In this case, it is necessary to face a significant disadvantage of polymer products due to possible and very common colonization of the surface by various microorganisms that can pose a potential danger to the patient. One of the possible solutions is to prepare polymer with antibacterial/antimicrobial properties that is resistant to bacterial colonization. The aim of this study was to contribute to the development of antimicrobial polymeric material ideal for covering vascular implants with subsequent use in transplant surgery. Therefore, the complexes of polymeric substances (hyaluronic acid and chitosan) with silver nitrate or silver phosphate nanoparticles were created, and their effects on gram-positive bacterial culture of *Staphylococcus aureus* were monitored. Stages of formation of complexes of silver nitrate and silver phosphate nanoparticles with polymeric compounds were characterized using electrochemical and spectrophotometric methods. Furthermore, the antimicrobial activity of complexes was determined using the methods of determination of growth curves and zones of inhibition. The results of this study revealed that the complex of chitosan, with silver phosphate nanoparticles, was the most suitable in order to have an antibacterial effect on bacterial culture of *Staphylococcus aureus*. Formation of this complex was under way at low concentrations of chitosan. The results of electrochemical determination corresponded with the results of spectrophotometric methods and verified good interaction and formation of the complex. The complex has an outstanding antibacterial effect and this effect was of several orders higher compared to other investigated complexes.

## 1. Introduction

Bacterial infections represent one of the most serious complications in vascular surgery [1]. This is mainly due to the increase in the using of artificial vascular prostheses [2]. Surgical site infections affect 1%–10% of patients who undergo vascular operations [3–7]. *Staphylococcus aureus* belongs to the most common contagious pathogens associated with clinical infections in vascular surgery [8–11], especially methicillin-resistant *S. aureus* (MRSA), which is responsible for more than 50% of infections in vascular surgery procedures [10,11]. To prevent infections in vascular surgery, substances with antibacterial effects are often used. Binding of antibiotics (rifampin, gentamicin, amikacin, vancomycin, levofloxacin) to vascular prostheses, impregnated with collagen or gelatin, represents one of the possible solutions to the infection issue [12–14].

**Figure 1:**
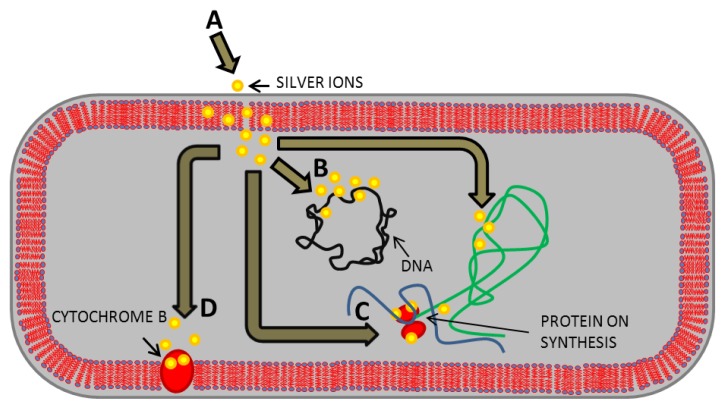
Mechanisms of action of the silver(I) ions on bacteria. (**A**) Silver(I) ions penetrate the bacterial cell wall and bind to the phospholipid layer of the cytoplasmic membrane; (**B**) Silver(I) ions bind the bacterial DNA with subsequent disrupting of DNA replication; (**C**) Silver(I) ions impair the ability of ribosomes to transcribe messenger RNA; (**D**) Silver(I) ions bind to the sulfhydryl group of the cytochrome b. Adopted and modified according to Ricco and Assadian [14].

From experimental studies to animal trials, rifampin bonded to vascular prosthesis Dacron has been determined to be the most effective prosthetic-antibiotic combination to date [13]. However, the use of antibiotics as an antimicrobial agent has one serious problem, the development of bacterial resistance to used antibiotics. Silver belongs to another promising substance with an antibacterial effect, which can reduce the number of infections after vascular surgery [15,16]. It is generally known that silver is highly toxic to microorganisms [17,18]. On the other hand, silver has a low toxicity to human cells, and a far lesser probability to cause bacterial resistance than antibiotics [19]. The mechanism of action of silver(I) ions on bacteria is based on inactivation of membrane proteins, binding with the bacterial DNA and disrupting DNA replication, impairing the ability of ribosomes to transcribe messenger RNA into the vital proteins required by the cell to function, inactivation of the cytochrome b (Figure 1), and consequent bactericidal activity [20,21]. Antimicrobial effects of silver can be increased by its application in the form of nanosilver [22]. Nanoparticles (NPs) with at least one dimension of 100 nm or less have unique physicochemical properties, such as high catalytic capabilities and ability to generate reactive oxygen species [23]. Silver, in the form of nanoparticles, could therefore be more reactive with its increased catalytic properties, and become more toxic for bacteria than silver(I) ions [24–27]. In searching for a proper antibacterial coating Pallavicini *et al.* prepared monolayers of silver nanoparticles (AgNPs) on glass surfaces [28]. AgNPs in the monolayers remained firmly grafted when the surfaces were exposed to water or phosphate saline buffer. About 15% silver release as silver(I) ions has been found after 15 days when the surfaces are exposed to water. The released silver cations were responsible of an efficient local antibacterial activity against *Escherichia coli* and *Staphylococcus aureus* bacterial strains. In another work, Taglietti *et al.* experiments on glutathione (GSH) coated silver nanoparticles (AgNPs) grafted on glass surface showed that the antibacterial activity against *Escherichia coli* and *Staphylococcus aureus* was reduced in comparison with uncoated AgNPs because of GSH coating and the limitation of the translational freedom of NPs [29].

Another way to increase the antimicrobial efficiency is to use silver in combination with other agents [30,31]. It is therefore important to investigate the effects of silver(I) ions and silver nanoparticles with compounds that promote their antibacterial effect. Both hyaluronic acid and chitosan belong to biopolymers with antimicrobial activity, which are characterized by biodegradability and biocompatibility with human body [32]. Hyaluronic acid is a high molecular weight glycosaminoglycan, generally regarded as an extracellular matrix component that facilitates cell locomotion and proliferation [33,34]. Hyaluronic acid is present in almost all biological fluids and tissues [34]. Chitosan is a linear polysaccharide composed of randomly distributed β-(1–4)-linked d-glucosamine and *N*-acetyl-d-glucosamine and is the most important derivative of chitin [35]. Chitosan is an effective material for biomedical applications because of its wound healing ability, antimicrobial, and anti-inflammatory activities [36–38]. Chitosan is able to bind metals and create complexes with them [37,39,40]. These interactions proceed through binding on amino groups of chitosan via chelation or complexation mechanism [35]. Similarly, it has been shown that hyaluronic acid is able to bind silver nanoparticles [41].

A combination of different forms of silver and biopolymer substances to cover the vascular prostheses is therefore a highly suitable solution in the fight against resistant strains of bacteria. The purpose of this study was to investigate the antimicrobial effect of silver(I) ions and silver phosphate nanoparticles (SPNPs), in combination with hyaluronic acid and chitosan.

## 2. Results and Discussion

Resistance to antibiotics represent a significant health issue. Modern biotechnological tools, based on the nanotechnological fundamentals, bring new possibilities and opportunities to overcome resistance. Synthesized nanoparticles and nanoparticles placed in nanotransporters enhance therapeutic possibilities and have sufficient effects against resistance induced by enzymatic pathways [42–44]. Effects of both silver(I) ions and silver-based NPs are sufficiently known; however, their combinations with various substances could improve technological possibilities in the treatment of serious diseases but is not fully understood and explored. In this study, we prepared silver phosphate nanoparticles (SPNPs) by a simple chemical synthesis. Formation of colloidal nano-Ag_3_PO_4_ was indicated by an immediate change of solution color to yellow, and the solution was opaque and turbid. The mixture was stirred for one hour and stored in the dark at 4 °C for further experiments. In addition, nanoparticles were characterized by SEM microscopy.

### 2.1. Characterization of Silver Phosphate Nanoparticles (SPNPs)

**Figure 2:**
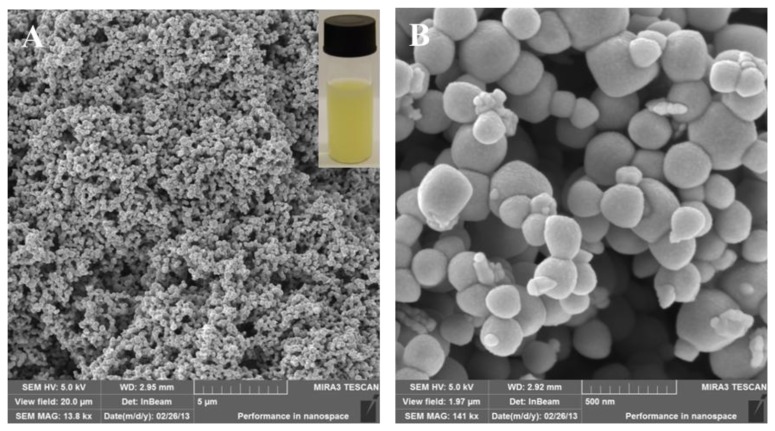
Silver phosphate nanoparticles (SPNPs) characterized by SEM and visually (**A**) SEM HV: 5 kV, view field: 20 μm, SEM MAF: 13.8 kx, WD: 2.95 mm, det: InBeam. In the upper right corner is a photo of the yellow colloid SPNPs solution; (**B**) SEM HV: 5 kV, view field: 1.97 μm, SEM MAF: 141 kx, WD: 2.92 mm, det: InBeam.

We have used a well-known precipitation reaction to prepare silver phosphate nanoparticles (SPNPs), as it has been shown in previously published papers [44,45]. Diluted solutions were used to prevent formation of bulky precipitate. The formation of SPNPs is very well observed by the turn of colorless solution to yellow, and colloid is formed (Figure 2A). Prepared SPNPs were characterized by SEM microscopy (Figure 2). Arising nanoparticles form a compact structure, which can be followed by formation of aggregates. Nevertheless, significant deep fissures occur in this structure. It may result in the binding of various molecules, eventually bacteria, in this structure (Figure 2A). Detailed SEM records show that SPNPs are spherical in shape and particle size varies in the 80–350 nm range. Minute nanoparticles that grow from bigger spherical SPNP are also visible (Figure 2B). Detailed microscopic study revealed that the majority of nanoparticles were between 200 and 300 nm in diameter (more than 80% of all SPNPs). This variation in nanoparticle size can be explained in terms of diffusion-limited aggregation and/or reaction-limited aggregation [46]. In less concentrated solution, reaction limited aggregation dominates and the cluster-cluster repulsion has to be overcome by a thermal activation process [47]. The nanoparticles were synthesized in the experiment and characterized three times and the results were repeatable.

### 2.2. Formation of Complexes of Silver with Polymeric Compounds

**Figure 3:**
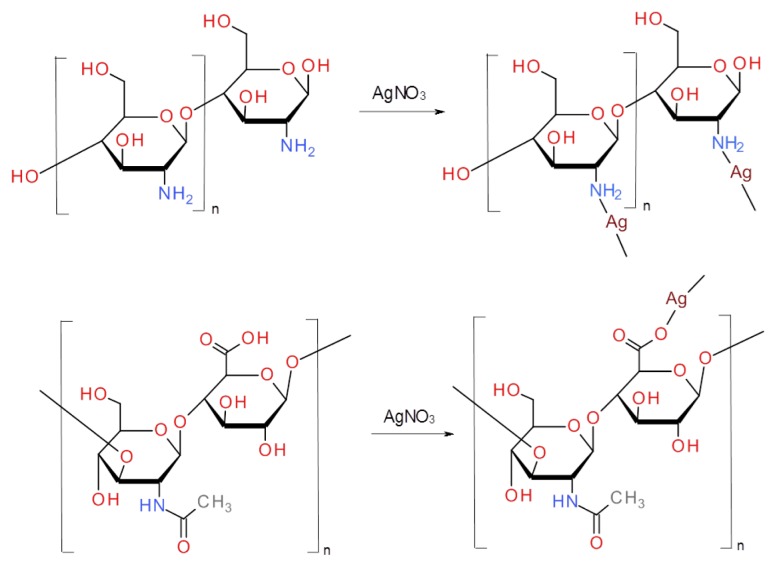
Suggested structures of silver complexes. Upper part of scheme represents complex of silver and chitosan, lower part stands for complex of hyaluronic acid and silver.

Prepared nanoparticles were used in further experiments for the monitoring and studying of their interactions with polymer compounds. Chitosan complexes with silver nitrate were prepared by addition of different concentrations of AgNO_3_ to chitosan. In the mild conditions, *i.e*., room temperature and short reaction time, only coordination of silver ions to electron pairs situated on chitosan nitrogens of amine groups can be expected. Proposed bonding of silver ions is shown in Figure 3. Addition of reducing agents or higher temperatures should lead to formation of silver nanoparticles as was observed by Bin Ahmad *et al.* [48]. Similarly, hyaluronic silver complex was formed after the addition of silver nitrate to the solution of hyaluronic acid. Ionic bond of carboxylic group with silver ion is the most probable mechanism in this case (Figure 3). There is also a possibility of the formation of chelate between two oxygens of carboxylic groups and silver ion, and binding to carboxylic group of second hyaluronic acid molecule. Coordination of silver ions to carboxylic groups is described by Abdel-Mohsen *et al.* [41]. The authors also used hyaluronic acid as a reducing agent and described reduction of silver ions into silver nanoparticles. They found that the reduction was dependent on pH and temperature. As we used only mild conditions, we cannot expect the formation of silver nanoparticles in our study. Formation of silver chitosan and hyaluronic complexes was confirmed by spectrophotometrical and electrochemical studies and was discussed later.

### 2.3. Characterization of Creation of Complex between Silver and Polymeric Compounds Using Electrochemical Methods

**Figure 4:**
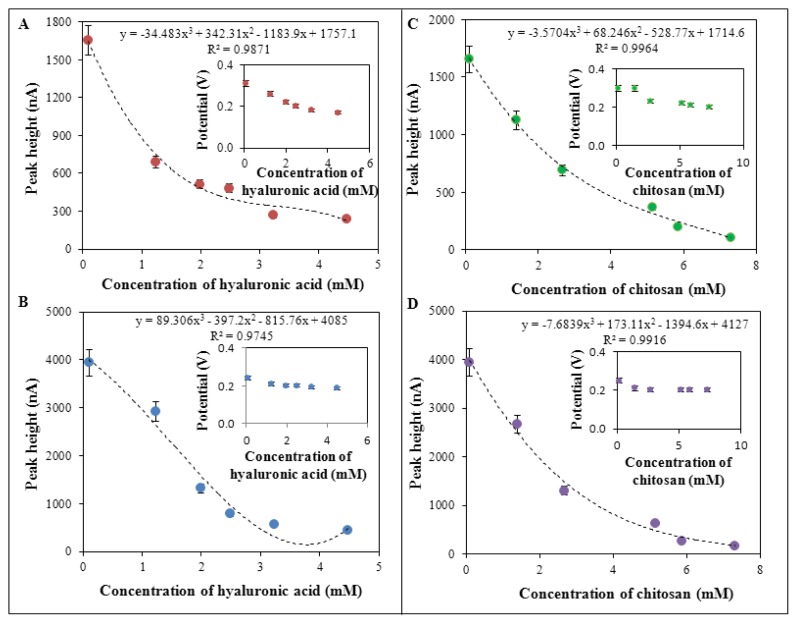
Electrochemical analysis of the interactions of: (**A**) hyaluronic acid (1.24, 1.99, 2.49, 3.23, 4.48 mM) and AgNO_3_ (100 μM); (**B**) hyaluronic acid (1.24, 1.99, 2.49, 3.23, 4.48 mM) and SPNPs (100 μM); (**C**) chitosan (1.4, 2.66, 5.14, 5.86, 7.32 mM) and silver ions AgNO_3_ (100 μM) or (**D**) chitosan (1.4, 2.66, 5.14, 5.86, 7.32 mM) and SPNPs (100 μM) using differential pulse voltammetry (DPV). Heights and potentials of silver(I) were determined and are shown. DPV parameters were as follows: start potential −0.2 V, end potential 0.5 V, amplitude 0.05 V, time of accumulation 60 s, acetate buffer pH 5.

The course of creation of complex of silver(I) ions and SPNPs with polymeric compounds was characterized firstly by electrochemical methods, namely differential pulse voltammetry, using the methodologies, which have been published in our previous papers [49–51]. Silver(I) ions (in the form of AgNO_3_) only provided a signal at the potential of 0.30 V and height of the signal came up to 1700 nA. On the other hand, silver phosphate nanoparticles (SPNPs) provided a signal at the potential of 0.25 V with a signal height of 4000 nA. AgNO_3_ and SPNPs were used at a concentration of 100 μM. Dependences of the signal potentials and signal heights on the concentrations of the compounds tested in experiments were evaluated based on the obtained results (Figure 4). The figure shows that increasing concentrations of hyaluronic acid or chitosan in combination with silver ions or SPNPs led to a decrease in height of electrochemical signal and it shifted into negative potential values (Figure 4).

In the case of a complex of hyaluronic acid with silver nitrate, the peak potential shifted from the potential of 0.30 V to 0.19 V (Figure 4A). In the case of complex of hyaluronic acid and SPNPs, signal was shifted from the potential of 0.25 V to 0.19 V (Figure 4B). Similar results were observed in the complex of chitosan and silver(I) ions as silver nitrate (shift from the potential of 0.30 V to 0.20 V, Figure 4C), and in complex of chitosan and SPNPs (shift from the potential of 0.25 V to 0.20 V, Figure 4D). The dependences of the height of signal on the concentration of applied compounds (hyaluronic acid and chitosan), in the combination with silver ions and SPNPs, show proportional decrease in the height of the signal with the increasing concentrations of the compounds of interest. These results indicate creation of complexes in all combinations of hyaluronic acid or chitosan with silver nitrate or SPNPs. The complexes of polymeric compounds (hyaluronic acid or chitosan) with silver particles (AgNO_3_ or SPNPs) were confirmed using different types of electrochemistry such as cyclic voltammetry in other studies [52,53].

### 2.4. Spectrophotometric Characterization of Course of Creation of Complexes of Silver and Polymeric Compounds

**Figure 5:**
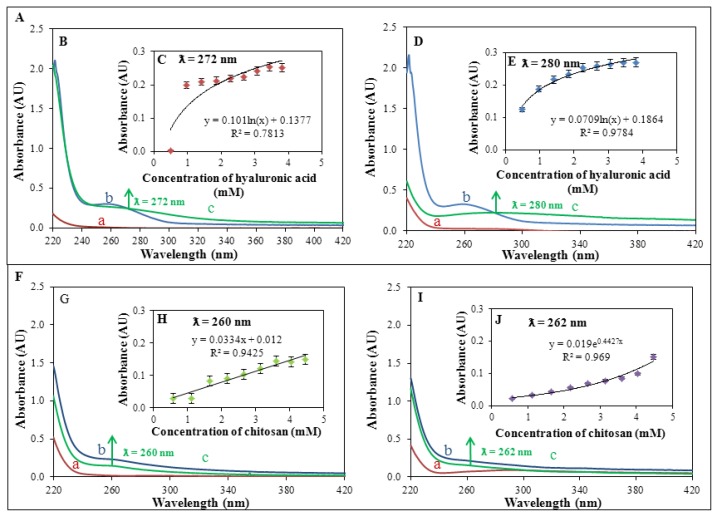
(**A**) Dependence of creation of a complex on applied concentration of hyaluronic acid (HA); (**B**) Spectral analysis in the range of 220–420 nm, a = 100 μM AgNO_3_, b = 24.9 mM HA and c = complex of 24.9 mM HA and 100 μM AgNO_3_, λ = 272 nm; (**C**) Dependence of creation of a complex (HA + 100 μM AgNO_3_) on applied concentration of HA, λ = 272 nm; (**D**) Spectral analysis within the range of 220–420 nm, a = 100 μM SPNPs, b = 24.9 mM HA and c = complex of 24.9 mM HA and 100 μM SPNPs, λ = 280 nm; (**E**) Dependence of creation of a complex (HA + 100 μM SPNPs) on applied concentration of HA, λ = 280 nm; (**F**) Dependence of creation of a complex on applied concentration of chitosan (CH); (**G**) Spectral analysis in the range of 220–420 nm, a = 100 μM AgNO_3_, b = 29.3 mM CH and c = complex of 29.3 mM CH and 100 μM AgNO_3_, λ = 260 nm; (**H**) Dependence of creation of a complex (CH + 100 μM AgNO_3_) on applied concentration of CH, λ = 260 nm; (**I**) Spectral analysis in the range of 220–420 nm, a = 100 μM SPNPs, b = 29.3 mM CH and c = complex of 29.3 mM CH and 100 μM SPNPs, λ = 262 nm; (**J**) Dependence of creation of a complex (CH + 100 μM SPNPs) on applied concentration of CH, λ = 262 nm.

Creation of complexes of silver and polymeric compounds was also studied by UV-VIS spectrophotometry in the wavelength range of 220–420 nm (Figure 5). UV/VIS spectrophotometry represents a simple and valuable method to study interactions and to characterize antimicrobial properties of various compounds [54,55]. This method confirmed results, obtained by the use of electrochemical methods. Firstly, absorption spectra of individual silver compounds were recorded (100 μM AgNO_3_ and 100 μM SPNPs). These compounds have no absorption maxima in the range 230–420 nm, see Figure 5B-a (100 μM AgNO_3_) and Figure 5D-a (100 μM SPNPs). The most distinct absorption maximum (at 260 nm) was recorded for 24.9 mM hyaluronic acid (see Figure 5B-b,D-b). Chitosan (29.3 mM) demonstrated absorption maximum at the same wavelength (260 nm). On the other hand, it was significantly less compared to hyaluronic acid (see Figure 5G-b,I-b). In the next step, polymeric compounds were mixed with silver accordingly. 100 μM solution of AgNO_3_/SPNPs was mixed with stock solution of hyaluronic acid (24.9 mM) to create final concentrations 0.5, 1.0, 1.4, 1.8, 2.3, 2.7, 3.1, 3.4, and 3.8 mM of hyaluronic acid. Absorption spectrum in the range 220–420 nm was recorded after each addition of hyaluronic acid. The same procedure was used for chitosan (concentrations 0.6, 1.1, 1.7, 2.2, 2.7, 3.1, 3.6, 4.0, and 4.5 mM). Creation of complexes of silver with polymeric compounds was accompanied by a change in absorption at different wavelengths, showed in Figure 5B-c (100 μM AgNO_3_ and 24.9 mM hyaluronic acid), Figure 5D-c (100 μM SPNPs and 24.9 mM hyaluronic acid), Figure 5G-c (12 mM AgNO_3_ and 29.3 mM chitosan), and Figure 5I-c (100 μM SPNPs and 29.3 mM chitosan). Titration of polymeric compounds (0.5, 1.0, 1.4, 1.8, 2.3, 2.7, 3.1, 3.4, and 3.8 mM hyaluronic acid or 0.6, 1.1, 1.7, 2.2, 2.7, 3.1, 3.6, 4.0, and 4.5 mM chitosan) with 100 μM AgNO_3_ or 100 μM SPNPs resulted in an increase in absorbance in created complexes, see Figure 5C (100 μM AgNO_3_-hyaluronic acid complex, λ = 272 nm), Figure 5D (100 μM SPNPs-hyaluronic acid complex, λ = 280 nm), Figure 5H (100 μM AgNO_3_-chitosan complex, λ = 260 nm), and Figure 5J (100 μM SPNPs-chitosan complex, λ = 262 nm). Complexes had their absorption maxima at different wavelengths compared to individual reagents. This fact confirms creation of the complexes. Only complex 100 μM AgNO_3_-chitosan had absorption maximum at the same wavelength (260 nm) as chitosan.

Electrochemical study (chapter 3.4) of this complex showed that creation of complex might also be expected in this case, although spectrophotometric measurement is not supporting this statement. Created complexes also differed in dependence of change of absorbance on the applied polymeric compound at the titration. First complex (100 μM AgNO_3_-hyaluronic acid) showed logarithmic dependence (*R*^2^ = 0.78), the second one (100 μM SPNPs-hyaluronic acid) logarithmic dependence too (*R*^2^ = 0.98), the third one (100 μM AgNO_3_-chitosan) linear dependence (*R*^2^ = 0.94), and the last studied complex (100 μM SPNPs-chitosan) exponential dependence (*R*^2^ = 0.97). Low concentrations of silver (0–100 μM, both AgNO_3_ and SPNPs) provided no signal in the VIS area. Due to this fact, created complexes were studied in the UV area of spectrum (260–280 nm). Nanosilver absorbs radiation in the VIS area (400 nm) in concentrations higher than 100 μM as is shown in the work of Abdel-Mohsen *et al.* [41], who used complex of chitosan and silver nanoparticles for the production of antimicrobial textiles. Nowadays, there are many applications using chitosan and silver as an antibacterial agent [41,54–56]. There are also mentioned complexes of silver and hyaluronic acid [41,48]. Our study provides the characteristics of this complex and presents potential use in clinical practice.

### 2.5. Mass Spectrometric Characterization of the Used Bacterial Strain

**Figure 6:**
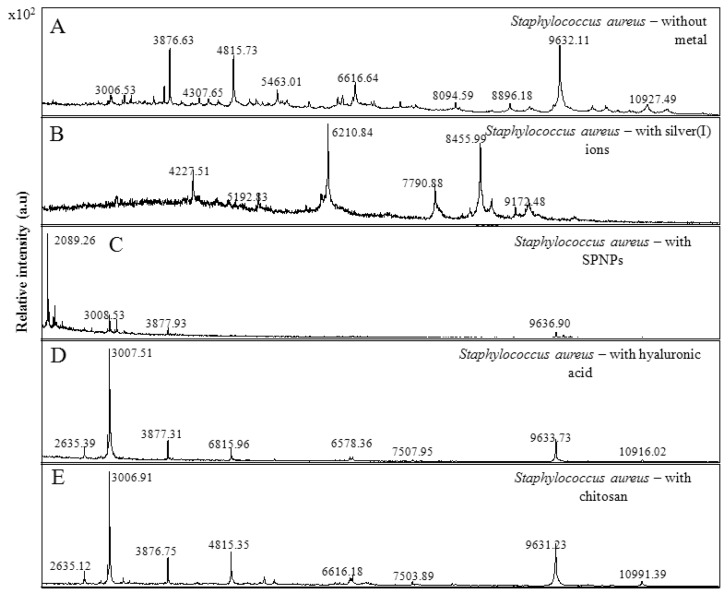
Matrix-assisted laser desorption-ionization time-of-flight (MALDI-TOF) mass spectra protein fingerprints for the identification of *Staphylococus aureus* (**A**) without metal, (**B**) with silver ions, (**C**) with SPNPs, (**D**) with hyaluronic acid and (**E**) with chitosan. Data were collected in the *m*/*z* 2000–20000 range after processing of 1 mL of culture of *S. aureus*.

We verified using matrix-assisted laser desorption-ionization time-of-flight (MALDI-TOF) technique for the presence of the complexes of interest in bacterial culture, which was treated with the abovementioned complexes in the following part of the study. MALDI-TOF is a technique that combines a soft, matrix-assisted, ionization process, and a TOF analyser to separate the generated ions [57,58]. In MALDI-TOF mass spectra, the mixture of biological samples with an energy-absorbing matrix allows the genesis of mostly intact-single-charged biomolecules. This tool is routinely used to identify bacterial species in clinical samples [59–61] and has been extensively used in biology to search biomarkers and to monitor protein post-translational modifications [62]. MALDI-TOF mass spectra were applied also for the classification of *S. aureus* strains. Obtained mass MALDI-TOF spectra of used bacterial culture of *S. aureus* are shown in Figure 6A. MALDI-TOF MS-based identification has been shown to be a fast and accurate technology in the identification of a variety of *S. aureus* strains [63–66]. Moreover, it has been found that the application of silver(I) ions to the bacterial culture led to a significant change in the mass spectrum of distinct molecular fragments of weight 4227 Da, 6210 Da, and 8455 Da (Figure 6B). The changes in the mass spectrum were also observed by the applications of SPNPs, hyaluronic acid, and chitosan to the bacterial culture (Figure 6C–E, respectively).

### 2.6. Determination of Antimicrobial Activity of Complexes of Silver and Polymeric Compounds

The results of the electrochemical and spectrophotometric determination indicate the creation of complexes of silver particles with polymeric compounds. Further, various combinations of polymers and silver particles were tested for their antimicrobial activity, as might be expected based on the previously published works [67–71]. Testing of antimicrobial activity of polymeric compounds (hyaluronic acid and chitosan) in complexes with silver and silver nanoparticles was chosen specifically due to their compatibility with human tissues (biocompatibility) and biodegradability [72–75].

**Figure 7:**
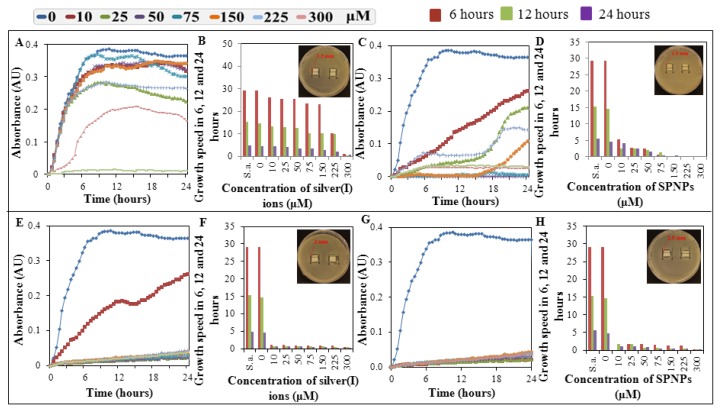
Growth curves, growth rate and inhibition zones of *Staphylococcus aureus* cultures after application of complex of silver ions (AgNO_3_) or silver phosphate nanoparticles (SPNPs) in various concentrations with hyaluronic acid (8.3 mM) or chitosan (9.7 mM). Growth curves (**A**) and growth rate (**B**) -applied hyaluronic acid with AgNO_3_, growth curves (**C**) and growth rate (**D**) -applied hyaluronic acid with SPNPs, growth curves (**E**) and growth rate (**F**) -applied chitosan with AgNO_3_, growth curves (**G**) and growth rate (**H**) -applied chitosan with SPNPs. In the upper right corner of the Figure 7B,D,F,H are photos of inhibition zones of the highest applied concentrations of silver(I) ions or SPNPs in complexes with hyaluronic acid or chitosan.

Polymeric compound chitosan was chosen due to its nontoxic, biocompatible, and biodegradable properties used for the targeted therapy, respectively for targeting of toxic compounds to specific cells and tissues in the human body [38]. Polymeric structure of studied compounds provides easy creation of complexes with metal ions [37,39,40]. Surprisingly, these complexes show higher antimicrobial properties than individual compounds. Testing of compounds and complexes was performed by the use of basic microbiological methods, method of determination of growth dependences (“growth curves”) [76–78], where the minimal concentrations of tested compounds inducing inhibition of growth-minimum inhibitory concentration (MIC) [79] and total inhibitory concentration (TIC) [80] are determined, or method based on the determination of inhibition zones [81–83]. Measuring inhibition zones was the first test to the antimicrobial properties of the compounds of interest. The first results were obtained after a 24 h long cultivation (Figure 7A–H). Only the hyaluronic acid or chitosan demonstrated no inhibitory effect in applied concentrations. Addition of silver ions (AgNO_3_) or silver phosphate nanoparticles (SPNPs) led to the significant increase in the inhibition of growth of culture of *S. aureus*. The greatest inhibitory effect had the combination of 9.7 mM chitosan with 300 μM silver(I) ions or 300 μM silver phosphate nanoparticles (Figure 7E,G). Size of inhibition zones for complex of chitosan and silver phosphate nanoparticles was about 2.5 mm, in the variant chitosan-silver ions these zones were slightly smaller and reached app. 2 mm. The smallest zones of inhibition were determined for the variants of 8.3 mM hyaluronic acid with 300 μM silver(I) ions and 300 μM silver phosphate nanoparticles (Figure 7A,C). Combination of hyaluronic acid with silver ions induced inhibition zones of app. 1.3 mm, the variant of hyaluronic acid with silver phosphate nanoparticles demonstrated inhibition zones as large as 1.5 mm.

To compare the results obtained by the method of determination of inhibition zones, growth characterization and determination of growth curves were used. This method clearly showed the highest antibacterial activity of the variants of silver ions and silver phosphate nanoparticles with chitosan (Figure 7E,G). Minimum inhibitory concentration (MIC) of 10 μM was determined for complex of chitosan and silver(I) ions, and total inhibitory concentration (TIC) was 25 μM and higher. In comparison, TIC for silver phosphate nanoparticles was determined as 10 μM. Complexes of silver and hyaluronic acid did not demonstrate significant antimicrobial effect. TIC for complex of silver phosphate nanoparticles and hyaluronic acid was determined as 150 μM, *i.e*., that this and higher concentrations (namely 150, 225, and 300 μM) led to the total growth inhibition of bacterial culture. On the other hand, complex of silver(I) ions with hyaluronic acid showed only moderate antimicrobial activity with TIC induced by concentration of 225 μM. Lower concentrations showed only slight antimicrobial activity, respectively only slight inhibition of growth of bacterial culture.

In addition, growth was evaluated at three times—6, 12, and 24 h. These results corresponded with previously obtained results and showed inhibition of growth of bacterial culture after application of studied compounds (Figure 7B,D,F,H). To analyse contribution of Ag form, other compounds (chitosan and hyaluronic acid) and time of treatment, a general regression model was used. Using this approach, results provide evidence that both forms of silver, selection of other compound (chitosan and hyaluronic acid) and combination of these affect the toxicity significantly (at *p* < 0.001). Inhibition concentrations are summarized in Table 1. In contrast, variable time does not provide significance. To provide more detailed analysis of obtained data, Turkey’s *post hoc* test for homogenous groups was performed (Table 2). Two distinct clusters are evident. First includes chitosan with both silver ions and silver phosphate nanoparticles, and hyaluronic acid with silver phosphate nanoparticles. The second cluster comprises the combination of hyaluronic acid with silver(I) ions. This combination provides distinctly lower cytotoxicity compared to those previously mentioned.

### 2.7. Biochemical Array

Biochemical arrays are used for characterization of bacterial strains of *Staphylococcus*, *Micrococcus* and *Stomatococcus*. These arrays (tests) focus changes in metabolism of chosen sugars in bacteria [84,85]. No significant changes in biochemical parameters, eventually moderate increase in enzymatic activity (for 10%–20%), were observed after application of hyaluronic acid (8.3 mM) to culture of *S. aureus* (0.1 OD). Application of 10 mM SPNPs led to decrease of metabolic activity for about 30%, compared to the control, untreated culture. In the case of the application of chitosan (29.3 mM) and 10 mM SPNPs we observed substantial decrease in metabolic activity of bacterial culture. Only minute changes in enzymatic activities (for 1%–5% of bacterial culture) of urease, phosphatase, β-glucosidase, and in metabolism, maltose and mannose were observed (not shown). Obtained results confirmed significant effect of complex of chitosan and silver phosphate nanoparticles on metabolic activity of bacterial culture of *Staphylococcus aureus*.

## 3. Experimental Section

### 3.1. Chemicals

Chemicals used in this study (AgNO_3_, Na_2_HPO_4_·7H_2_O, hyaluronic acid, chitosan, tryptone, yeast extract, NaCl) were purchased from Sigma-Aldrich (St. Louis, MO, USA) in ACS purity unless noted otherwise. Deionized water was prepared using reverse osmosis equipment Aqual 25 (Brno, Czech Republic). Deionized water was further purified by using a MiliQ Direct QUV apparatus equipped with a UV lamp. The resistance was 18 MΩ. The pH was measured using pH meter WTW inoLab (Weilheim, Germany).

### 3.2. Knitted Vascular Prosthesis

Vascular prostheses are made of polyester fibres, using a knitting technology, and are covered on the marked side with a continuous layer of bovine collagen type I. These vascular prostheses were made by Research Institute of Knitting Technologies in Brno (Brno, Czech Republic) of different lengths and thicknesses, usually marked as Ra 1v K. Vascular prostheses are solid, flexible, do not fray, and are provided with a color guide line. Water permeability through the wall of the vascular prosthesis is 0–5 mL cm^−2^ min^−1^. Vascular prostheses are sterilized by irradiation and are not designed for resterilization.

### 3.3. Preparation of Silver Phosphate Nanoparticles (SPNPs)

SPNPs were prepared by the method of Khan *et al.* [47]. Heptahydrate of disodium hydrogen phosphate (0.134 g) was dissolved in ACS water (25 mL). A solution of AgNO_3_ (0.085 g) in 25 mL of ACS water was quickly added to a stirred solution of disodium hydrogen phosphate. There was an immediate change of solution colour to yellow with formation of colloidal nanoform of Ag_3_PO_4_. The mixture was stirred for 1 h. SPNPs were stored in the dark at 4 °C. One millilitre of mixture contains 1.08 mg of Ag. To create complexes, stock solutions of silver(I) ions (AgNO_3_—12 mM), silver phosphate nanoparticles (SPNPs—10 mM), and a pair of polymer substances, hyaluronic acid (24.9 mM) and chitosan (29.3 mM) were used.

### 3.4. Cultivation of Staphylococcus aureus

*Staphylococcus aureus* (NCTC 8511) was obtained from the Czech Collection of Microorganisms, Faculty of Science, Masaryk University, Brno, Czech Republic. Strains were stored in the form of a spore suspension in 20% (*v*/*v*) glycerol at −20 °C. Prior to use, the strains were thawed and the glycerol was removed by washing with distilled water in this study. The composition of cultivation medium was as it follows: meat peptone 5 g L^−1^, NaCl 5 g L^−1^, bovine extract 1.5 g L^−1^, yeast extract 1.5 g L^−1^ (HIMEDIA, Mumbai, India), sterilized MilliQ water with 18 MΩ. pH of the cultivation medium was adjusted at 7.4 before sterilization. Sterilization of media was carried out at 121 °C for 30 min in sterilizer (Tuttnauer 2450EL, Beit Shemesh, Israel). The prepared cultivation media were inoculated with bacterial culture into 25 ml Erlenmeyer flasks. After the inoculation, bacterial cultures were cultivated for 24 h on a shaker at 600 rpm and 37 °C. Bacterial culture cultivated under these conditions was diluted by cultivation medium to OD600 = 0.1 and used in the following experiments.

### 3.5. Determination of Antibacterial Properties

To determine the antimicrobial effect of silver(I) ions and silver phosphate nanoparticles (SPNPs) in complexes with hyaluronic acid and chitosan, the measurement of the inhibition zones was performed. Agar surface in Petri dish was covered with a mixture of 100 mL of 24 h culture of *S. aureus* in the exponential phase of growth, and 3 mL of LB medium (Luria Bertani medium). Excess volume of the mixture of the Petri dishes was aspirated. Squares of size of 1 × 1 cm were cut out from the knitted vascular prosthesis. These squares were mixed with combinations of hyaluronic acid or chitosan with different concentration of silver(I) ions or silver phosphate nanoparticles in Eppendorf tubes. Soaked squares were then laid crosswise on a Petri dish, two squares per dish. Petri dishes were insulated against possible external contamination and placed in a thermostat (Tuttnauer 2450EL, Beit Shemesh, Israel) set at 37 °C for 24 h. After 24 h of incubation, the inhibition zones were measured and photographed in each Petri dish.

### 3.6. Determination of Growth Curves

The second procedure for the evaluation of an antimicrobial effect of tested compounds and their combinations was based on the measurement of an absorbance of these compounds in combination with a bacterial culture of *Staphylococcus aureus*. An apparatus Multiskan EX (Thermo Fisher Scientific, Bremen, Germany) via Ascent Software for Multiskan, and subsequent analysis in the form of growth curves, was used. The same culture as in the measurement of inhibition zones was diluted with LB medium to absorbance of 0.1 measured using a Specord spectrophotometer 210 (Analytik Jena, Jena, Germany) at a wavelength of 600 nm. The diluted culture was pipetted into a microplate (total volume of 300 μL) alone as a control variant, or with various concentrations of tested substances (silver(I) ions or SPNPs). The concentration of silver(I) ions and SPNPs were 0, 10, 25, 50, 75, 150, 225, and 300 μM, the concentration of hyaluronic acid (8.3 mM) and chitosan (9.7 mM) were always the same. Measurements were carried out at time 0, then each half-hour for 24 h at 37 °C, at a wavelength of 620 nm. The measured absorbance values were analysed in graphic form as growth curves for each experimental group individually.

### 3.7. Biochemical Tests

Bacterial culture was treated with individual substances deposited on the bottom of the microplate wells. The substances were as follows: urease, arginine, ornithine, β-galactosidase, β-glucuronidase, β-glucosidase, phosphatase, esculin, *N*-acetyl-β-d-glucosamine, sucrose, mannitol, xylose, galactose, trehalose, maltose, mannose, lactose, sorbitol, ribose, fructose, cellobiose, arabinose, xylitol, and raffinose. All substances were purchased from Sigma-Aldrich. Bacterial culture of *S. aureus* of the concentration about 8.0.107 KTJ/1 mL was pipetted into the individual wells. This culture was diluted to an absorbance of 0.1 measured using a Specord spectrophotometer set at a wavelength of 600 nm. Silver nanoparticles at a concentration of 10 μM and hyaluronic acid (24.9 mM) or chitosan (29.3 mM) were subsequently added to this culture. Complexes were measured for 24 h using a Multiskan EX apparatus (Thermo Fisher Scientific, Germany) via Ascent Software for Multiskan.

### 3.8. Determination of Protein Matrix-Assisted Laser Desorption-Ionization Time-of-Flight (MALDI) Mass Spectra

A sample of 500 μL *S. aureus* (0.1 OD) culture, cultivated overnight, was centrifuged at 14,000 × *g* for 2 min. After this, supernatant was discarded and the pellet was suspended in 300 μL of deionized water. Then, 900 μL of ethanol was added. After centrifugation at 14,000 × *g* for 2 min, supernatant was discarded and the obtained pellet was air-dried. The pellet was then dissolved in 25 μL of 70% formic acid (*v*/*v*) and 25 μL of acetonitrile and mixed. The samples were centrifuged at 14,000 × *g* for 2 min and 1 μL of the clear supernatant was spotted in duplicate onto the MALDI target (MTP 384 target polished steel plate; Bruker Daltonics, Bremen, Germany) and air-dried at room temperature. Then, each spot was overlaid with 1 μL of α-cyano-4-hydroxycinnamic acid (HCCA) matrix solution saturated with organic solvent (50% acetonitrile and 2.5% trifluoroacetic acid, both *v*/*v*) and air-dried completely prior to matrix-assisted laser desorption-ionization time-of-flight mass spectrometric (MALDI-TOF MS) measurement. Spectra were taken in the *m*/*z* range of 2000 Da to 20,000 Da, and each was a result of the accumulation of at least 1000 laser shots obtained from ten different regions of the same sample spot. Spectra were analysed with the Flex Analysis software (Version 3.4). Prior to analysis, the mass spectrometer was externally calibrated with a peptide mix of bombesin, angiotensin I, glu-fibrinopeptide B, adrenocorticotropic hormone (ACTH) (18–39), ubiquitine, and cytochrome c. Spectra with peaks outside the allowed average were not considered. Modified spectra were loaded into the MALDI BioTyper™ 3.1 Version (Bruker Daltonik GmbH, Bremen, Germany).

### 3.9. Electrochemical Determination of Silver(I) Ions, and SPNPs in Complexes with Hyaluronic Acid and Chitosan Using Sensor Array

Differential pulse voltammetric (DPV) measurements [86–88] were performed using a PalmSens (PalmSens, The Netherlands) potentiostat connected with sensor array (Brno University of Technology, Brno, Czech Republic) through a control box (Brno University of Technology). For smoothing and baseline correction the PalmSens software supplied was employed. As the supporting electrolyte, acetate buffer (0.2 M CH_3_COOH; 0.2 M CH_3_COONa) was used. Applied volume of samples was 50 μL. DPV conditions were as follows: start potential −0.2 V, end potential 0.5 V, amplitude 0.05 V, time of accumulation 60 s.

### 3.10. Spectrophotometric Determination of Silver(I) Ions, and SPNPs in Complexes with Hyaluronic Acid and Chitosan

Absorption spectra were recorded by a SPECORD 210 spectrophotometer (Analytic Jena, Jena, Germany) in the wavelength range λ = 200–700 nm using the WinAspect 2.2.7.0. (Analytic Jena, Jena, Germany). Quartz cuvettes of 1 cm optical path (Hellma, Essex, Great Britain) were used. The analyzed sample was made up of a combination of tested substances (12 mM silver(I) ions or 10 mM SPNPs with 24.9 mM hyaluronic acid or 29.3 mM chitosan). Primarily, we registered absorption spectra (200–700 nm) of each of reagents (AgNO_3_ and SPNPs). Then, a polymer substance of 24.9 mM hyaluronic acid or 29.3 mM chitosan in 20 μL volume was pipetted into the sample of 12 mM AgNO_3_ or 10 mM SPNPs. The samples were incubated for 5 min at 25 °C under vigorous stirring. Then, absorption spectra were recorded. This procedure was repeated eight times. ACS water was used as a solvent. Final concentrations of polymer substances were 0.5, 1.0, 1.4, 1.8, 2.3, 2.7, 3.1, 3.4, and 3.8 mM for hyaluronic acid and 0.6, 1.1, 1.7, 2.2, 2.7, 3.1, 3.6, 4.0, and 4.5 mM for chitosan.

### 3.11. Scanning Electron Microscope

Structures were characterized by scanning electron microscopy. For documentation of the selected nanomaterials, a FEG-SEM MIRA XMU (Tescan, Brno, Czech Republic) was used. This model is equipped with a high brightness Schottky field emitter for low noise imaging at fast scanning rates. SEM was fitted with Everhart-Thronley type of SE detector, high speed YAG scintillator based BSE detector, panchromatic CL Detector, and EDX spectrometer. The MIRA 3 XMU system is based on a large specimen chamber with motorized stage movements 130 × 130 mm. Samples were coated by 10 nm of carbon (Sigma-Aldrich, St. Louis, MO, USA) to prevent a sample charging. A carbon coater K950X (Quorum Technologies, Grinstead, UK) was used. For automated acquisition of selected areas, a TESCAN proprietary software tool Image Snapper was used. The software enables automatic acquisition of selected areas with defined resolution. Different conditions were optimized in order to reach either minimum analysis time or maximum detail during overnight, automated analysis. An accelerating voltage of 15 kV and beam currents of about 1 nA gives satisfactory results regarding maximum throughput.

### 3.12. Statistical Analyses

Software STATISTICA (data analysis software system), version 10 .0 (Tulsa, OK, USA) was used for data processing. Half-maximal concentrations (IC_50_) were calculated from logarithmic regression of sigmoideal dose-response curve. General regression model was used to analyse differences between the combinations of compounds. To reveal differences between cell lines, Turkey’s *post hoc* test within homogenous groups was employed. Unless noted otherwise, *p* < 0.05 was considered significant.

## 4. Conclusions

Testing of antibacterial properties of silver complexes (AgNO_3_ or SPNPs) with the body’s own substances (hyaluronic acid or chitosan) was carried out on the bacterial culture of *Staphylococcus aureus*, by measuring the inhibition zones and determination of growth characteristics. Our results indicate the highest antimicrobial properties of complexes of silver(I) ions and silver nanoparticles with chitosan. The results can be used for further experiments with possible application in vascular surgery, particularly to reduce bacterial infections, which represent a high risk in the implantation of artificial vascular grafts.

## Figures and Tables

**Table 1:** Half-maximal inhibition concentrations (IC_50_).

Type of Ag	Other compound	Time	IC_50_ (μM)
SPNPs	chitosan	6	5.10
SPNPs	chitosan	12	3.72
SPNPs	chitosan	24	1.00
AgNO_3_	hyaluronic acid	6	209.63
AgNO_3_	hyaluronic acid	12	246.55
AgNO_3_	hyaluronic acid	24	188.15
SPNPs	hyaluronic acid	6	1.00
SPNPs	hyaluronic acid	12	1.00
SPNPs	hyaluronic acid	24	29.53
AgNO_3_	chitosan	6	1.00
AgNO_3_	chitosan	12	1.00
AgNO_3_	chitosan	24	1.00

**Table 2:** Turkey’s post hoc test; mean IC_50_ (μM). Asterisks indicate distribution of complexes into two homogenous groups (1 and 2) at *p* < 0.05.

Number	Type of Ag	Other compound	IC_50_ (μM)-Mean	1	2
1	AgNO_3_	chitosan	1.0000	****	
2	SPNPs	chitosan	3.2704	****	
3	SPNPs	hyaluronic acid	10.5100	****	
4	AgNO_3_	hyaluronic acid	214.7771		****
